# Hyperopia Is Not Causally Associated With a Major Deficit in Educational Attainment

**DOI:** 10.1167/tvst.10.12.34

**Published:** 2021-10-28

**Authors:** Denis Plotnikov, Nuala A. Sheehan, Cathy Williams, Denize Atan, Jeremy A. Guggenheim, for the UK Biobank Eye and Vision Consortium

**Affiliations:** 1 School of Optometry & Vision Sciences, Cardiff University, Cardiff, UK; 2 Kazan State Medical University, Kazan, Russia; 3 Department of Health Sciences, University of Leicester, Leicester, UK; 4 Population Health Sciences, Bristol Medical School, University of Bristol, Bristol, UK; 5 Translational Health Sciences, Bristol Medical School, University of Bristol, Bristol, UK

**Keywords:** hyperopia, education, Mendelian randomization, genetic epidemiology, refractive error

## Abstract

**Purpose:**

Hyperopia (farsightedness) has been associated with a deficit in children's educational attainment in some studies. We aimed to investigate the causality of the relationship between refractive error and educational attainment.

**Methods:**

Mendelian randomization (MR) analysis in 74,463 UK Biobank participants was used to estimate the causal effect of refractive error on years spent in full-time education, which was taken as a measure of educational attainment. A polygenic score for refractive error derived from 129 genetic variants was used as the instrumental variable. Both linear and nonlinear (allowing for a nonlinear relationship between refractive error and educational attainment) MR analyses were performed.

**Results:**

Assuming a linear relationship between refractive error and educational attainment, the causal effect of refractive error on years spent in full-time education was estimated as −0.01 yr/D (95% confidence interval, −0.04 to +0.02; *P* = 0.52), suggesting minimal evidence for a non-zero causal effect. Nonlinear MR supported the hypothesis of the nonlinearity of the relationship (*I*^2^ = 80.3%; Cochran's *Q* = 28.2; *P* = 8.8e-05) but did not suggest that hyperopia was associated with a major deficit in years spent in education.

**Conclusions:**

This work suggested that the causal relationship between refractive error and educational attainment was nonlinear but found no evidence that moderate hyperopia caused a major deficit in educational attainment. Importantly, however, because statistical power was limited and some participants with moderate hyperopia would have worn spectacles as children, modest adverse effects may have gone undetected.

**Translational Relevance:**

These findings suggest that moderate hyperopia does not cause a major deficit in educational attainment.

## Introduction

In the first few months after they are born, the majority of infants have a low level of hyperopia (approximately +2.00 diopters [D], on average).^1^ Over the next 3 to 6 years, this hyperopia reduces in magnitude in most infants through the process of emmetropization. However, some infants do not emmetropize but instead maintain a relatively high degree of hyperopia throughout this period and on into adulthood.^2^ Children with hyperopia are prescribed spectacles either to provide clear vision as their accommodation is not effective enough to compensate for their hyperopia or to alleviate their eyestrain symptoms.^3^^–^^5^

Randomized controlled trials (RCTs) suggest that prescribing spectacles to 7- to 11-month-old infants with hyperopia of +3.50 D or more reduces the risk of strabismus and amblyopia.^6^^–^^8^ However, there is no consensus on whether prescribing spectacles to asymptomatic school-age children for lower levels of hyperopia improves their reading performance or educational attainment. Observational studies largely support the existence of an association between hyperopia and poorer reading skills or educational attainment, especially in *uncorrected* hyperopia,^9^^–^^16^ but not all studies have demonstrated such an association.^17^^–^^19^

**Figure 1.**
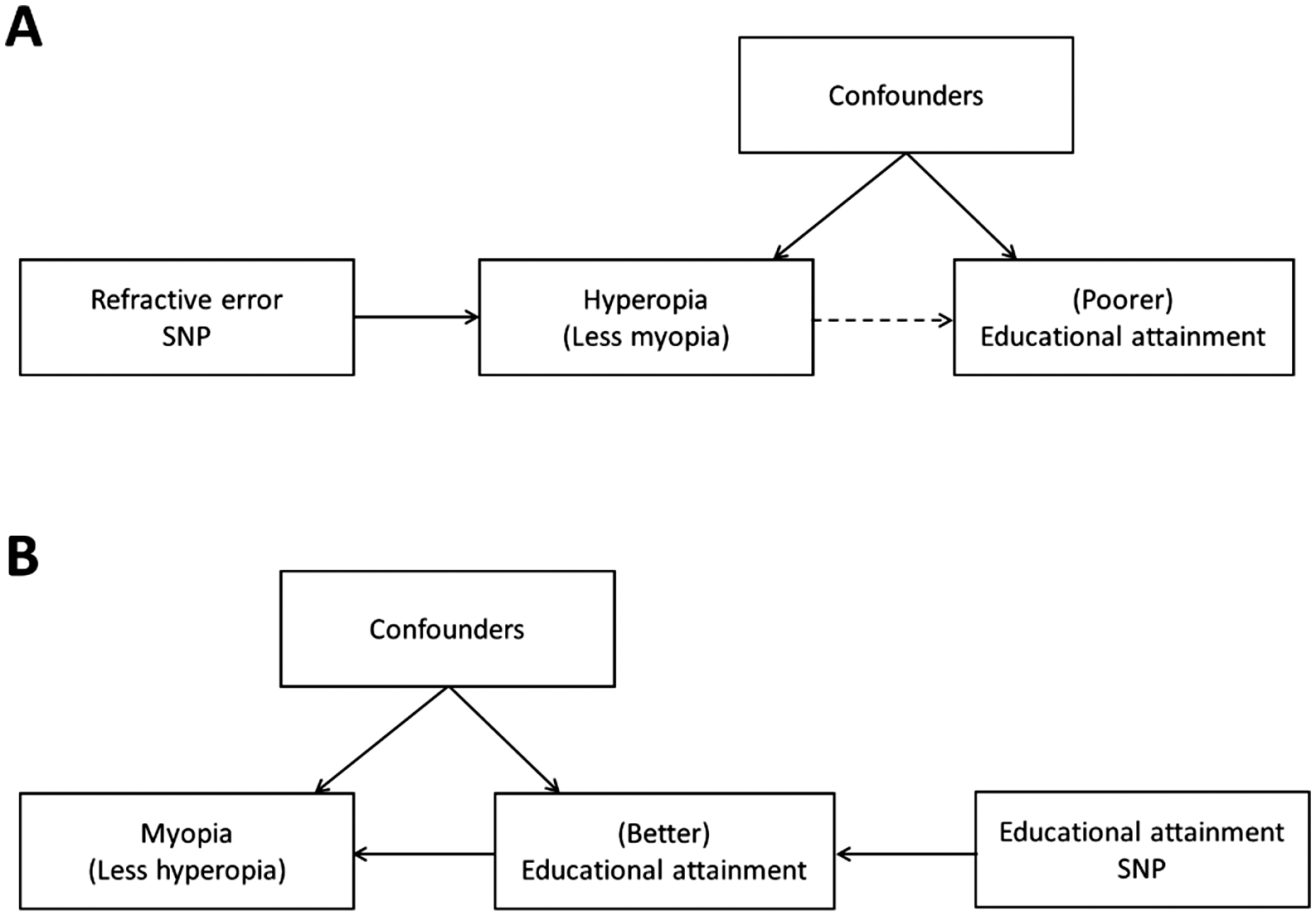
Investigating the causal relationship between refractive error and education. An association between refractive error (hyperopia or myopia) and educational attainment has been reported in several observational studies. One potential cause of this relationship is the action of a confounder such as socioeconomic status that influences both refractive error and educational attainment (A, B). A second potential cause of the relationship, as illustrated in (A), is that hyperopia causes (poorer) educational attainment. In the current work, we propose using genetic variants strongly associated with refractive error as instrumental variables in a Mendelian randomization analysis to test this hypothesis: If hyperopia is a cause of poorer educational attainment, then we would expect an association between the refractive error SNP genotypes and years spent in education. This association would, under certain assumptions (described in the main text), be free from bias due to confounders such as socioeconomic status. A third potential cause of the relationship is illustrated in (B). Previously, Mountjoy et al.^21^ used genetic variants strongly associated with educational attainment in a Mendelian randomization analysis and found evidence that better educational attainment causes myopia (or less hyperopia).

For children growing up in countries with well-developed education systems, fatigue of the accommodation system and imbalance of the accommodation–convergence mechanism are plausible explanations for the observational association between hyperopia and poorer educational attainment—that is, hyperopia produces accommodative fatigue-induced eyestrain, which deters children from performing near-viewing study tasks (Fig. 1A). A potential alternative explanation is that exposure to the educational environment causes a shift toward a more negative, less hyperopic refractive error (Fig. 1B). Consistent with this latter explanation, recent studies by Cuellar-Partida et al.^20^ and Mountjoy et al.^21^ using the technique of Mendelian randomization have reported that additional years spent in education are causally associated with a more negative refractive error. In addition, the study by Mountjoy et al.^21^ specifically tested for a role of refractive error on educational attainment (Fig. 1A) but found negligible support for a causal effect in this direction. The latter result argues against hyperopia playing a causal role in educational attainment and instead favors causation in the reverse direction as the more likely explanation for any observed association.

Mendelian randomization (MR) is a method for making causal inferences using observational data.^22^^–^^24^ It has become a popular approach in situations where RCTs are impractical or unethical or to provide preliminary support for a hypothesized causal relationship before investing in a costly RCT. MR exploits genetic variants associated with an exposure of interest as instrumental variables (IVs). For example, Cuellar-Partida et al.^20^ and Mountjoy et al.^21^ used single nucleotide polymorphisms (SNPs) associated with educational attainment as IVs to study the causal effect of education on refractive error. For a SNP to be a valid IV it must satisfy the three so-called instrumental variable assumptions that the IV must (1) be robustly associated with the exposure, (2) be independent of unmeasured confounding factors, and (3) have an effect on the outcome mediated solely through the exposure.^25^ Unlike the first assumption, the latter two assumptions cannot be confirmed experimentally, which has led to the development of numerous modifications of the standard MR approach that aim to provide valid inference even when these assumptions do not hold for a proportion of the IVs.^26^

In the MR study of Mountjoy et al.^21^ mentioned above, SNPs associated with refractive error were used as IVs to test for a causal effect of refractive error on the number of years spent in full-time education in a sample of UK Biobank participants. The causal effect estimate was very small, and the accompanying 95% confidence interval (CI) spanned the null effect of zero. Under a specific set of seven different assumptions, this result implies that hyperopia is not a cause of poor educational attainment. The first three assumptions are that the SNPs used as IVs (1) are associated with refractive error in childhood, (2) are independent of unmeasured confounding factors, and (3) have an effect on educational attainment mediated solely through refractive error. The additional assumptions are that (4) hyperopia present in adulthood would have been present in childhood, (5) years spent in full-time education is a valid proxy for educational attainment, (6) the fact that a proportion of children with hyperopia are prescribed spectacles to correct their potential visual deficit has no influence on the relationship between refractive error and educational attainment in the sample as a whole, and (7) the relationship between refractive error and educational attainment is linear.

The current work addresses the last of these assumptions—namely, that the relationship between refractive error and educational attainment is linear (later in the article, we also discuss the plausibility of the other assumptions). Support for the linearity assumption is currently lacking, and it seems credible that hyperopia and myopia may *both* hinder children's academic development, which would result in an inverse U-shaped relationship between refractive error and educational attainment rather than a linear relationship. Hence, we investigated this issue using the technique of nonlinear Mendelian randomization.

## Methods

### Study Cohort

The UK Biobank recruited approximately 500,000 participants 37 to 73 years of age between 2006 and 2010 through attendance at a series of assessment centers located across the United Kingdom.^27^^,^^28^ Ethical approval was obtained from the NHS Research Ethics Committee (11/NW/0382). Cross-sectional data from the baseline assessment visit were analyzed in this study. Quality control procedures followed those reported previously.^29^^–^^31^ An ophthalmic examination was included as part of the baseline visit physical assessment test battery from 2009 onward.^32^ Approximately 23% of participants underwent the ophthalmic assessment, which included non-cycloplegic autorefraction (RC 5000 autorefractor; Tomey, Aichi, Japan) performed after removal of spectacles or contact lenses. Repeat autorefraction measurements of the same eye were averaged after measurements flagged as unreliable were removed. Mean spherical equivalent refractive error was calculated as the spherical power plus half of the cylindrical power. Finally, the mean spherical equivalent refractive error was averaged for the two eyes of each individual.^33^ Participants reported a history of any eye disorders. Linked hospital records were also searched for a history of eye disorders.^21^ Using this information, we excluded participants with self-reported or hospital-recorded history of cataract or laser eye surgery, eye trauma resulting in vision loss, or corneal graft surgery.^21^ UK Biobank participants without a college or university degree were asked, “At what age did you complete your continuous full-time education?” Participants with a college or university degree were coded as having completed full-time education at age 21 years.^34^ Governed by the Education Act of 1870, school attendance in England and Wales is compulsory starting at age 5 years.^35^ Given the consistent starting age, age at education completion therefore provides a measure of years spent in full-time education. Following Okbay et al.,^36^ we labeled this variable “EduYears.”

UK Biobank participants were genotyped using either the Affymetrix UK BiLEVE Axiom array or the Affymetrix UK Biobank array (Affymetrix, Santa Clara, CA), which assessed approximately 850,000 genetic variants.^37^ The data underwent stringent quality-control checks, and additional genotypes were imputed by UK Biobank researchers.^37^ Participants reported their place of birth, which was coded as a geographical coordinate on a north–south axis (“Northing”) and an east–west axis (“Easting”). Individuals were excluded from the analyses if they self-reported their country of birth as a country other than England or Wales, if they had non-White British European genetic ancestry (as defined by Bycroft et al.^37^), or excessive genetic heterozygosity (more than 4 SDs from the mean). As a result of the vision assessment being introduced partway through recruitment, the majority of participants (99.3%) were seen at nine of the 22 UK Biobank assessment centers. Participants who attended an assessment center that recruited fewer than 500 individuals were also excluded. To avoid the inclusion of close relatives in the sample, the maximum set of individuals with a kinship coefficient no greater than expected for third cousins was generated, using the R package igraph (R Foundation for Statistical Computing, Vienna, Austria), following the method reported by Bycroft et al.^37^ After applying these exclusions, the final sample was comprised of 74,463 participants. Supplementary Figure S1 provides a flow chart detailing the participant selection and exclusion steps.

### Instrumental Variables for Mendelian Randomization

Tedja et al.^33^ reported 161 SNPs associated with refractive error at genome-wide statistical significance (*P* < 5 × 10^−8^) in a large genome-wide association study (GWAS) meta-analysis of studies by the CREAM consortium and the 23andMe Inc. personal genomics company. We elected to use SNPs from the above GWAS reported by Tedja et al.,^33^ rather than those reported more recently in a larger GWAS for refractive error,^38^ as the more recent GWAS sample included UK Biobank participants and therefore risked selecting a biased set of instrumental variables due to the winner's curse.^39^ Following the criteria adopted by Wood and Guggenheim,^40^ we excluded 12 SNPs that did not replicate in an independent sample of 95,505 UK Biobank participants of European ancestry, 18 SNPs in linkage disequilibrium (*r*^2^ > 0.05) with another variant in the set, and two SNPs with Hardy–Weinberg equilibrium test *P* < 0.05, leaving 129 SNPs to use in the downstream analyses (Supplementary Table S1). The functional roles of the 129 SNPs and thus the pathways through which they give rise to an association with refractive error are not known (accordingly, in view of the strong assumption that these SNPs are valid IVs, we describe below sensitivity analyses that are robust to violations of the IV assumptions). We constructed a weighted polygenic score (PGS) for refractive error using these 129 SNPs. A polygenic score is a single value quantifying a person's genetic predisposition to a specific trait or disease.^41^ The polygenic score was calculated according to the following equation:

$$\begin{array}{c} \mathrm{PGS}=\;\sum_{j=1}^{n}X_{j}\times \beta_{j} \end{array}$$

where PGS is the polygenic score for an individual, *X_j_* is the number of effect alleles of SNP*_j_* (0, 1, or 2) carried by the individual, β*_j_* is a weighting factor for SNP*_j_*, and *j* = 1,2, …, 129 indexes the SNPs. The weighting factors (SNP weights) quantifying the degree of association between the SNP and refractive error for the polygenic score were calculated as the square of the Z-score from the Stage-3 analysis of Europeans reported by Tedja et al.^33^ Note that, conventionally, SNP regression coefficients from a GWAS are used as SNP weights. However, SNP regression coefficients were not reported by Tedja et al. To justify our use of squared Z-scores in place of the conventionally used regression coefficient SNP weights, we examined the relationship between the squared Z-score SNP weights and regression coefficient SNP weights calculated in the UK Biobank analysis sample (Supplementary Fig. S2). This yielded a linear relationship, justifying our choice of squared Z-scores as SNP weights.

### Statistical Analyses

The observational association between refractive error and EduYears was estimated using linear regression in the sample of 74,463 UK Biobank participants, with adjustment for the potential confounders, age, age^2, age^3, gender, Northing, Easting, genotyping array, and the first 10 ancestry genetic principal components (PCs). Ancestry PCs and the geographical coordinates Northing and Easting were included to account for potential subtle population stratification effects present within the UK population.^42^

Linear MR analysis (i.e., MR assuming a linear relationship between the exposure and the outcome) was performed for the sample of 74,463 UK Biobank participants, with the polygenic score for refractive error as a single instrumental variable. Analysis was implemented using a limited information maximum likelihood estimator from the ivmodel R package.^43^ The model was adjusted for age, age^2, age^3, gender, Northing, Easting, genotyping array, and the first 10 ancestry PCs. The *F* statistic from the first-stage regression was used to assess the strength of the association between the polygenic score and refractive error.^44^ The statistical power of this analysis for a type-I error rate α = 0.05 was calculated using the IVpower function from the ivmodel R package.^43^ A confounding bias plot^45^ was used to assess covariate balance across the polygenic score instrumental variable and the exposure variable (refractive error).

To assess evidence for nonlinearity in the causal relationship between refractive error and education—such as a lower level of educational attainment restricted to individuals with hyperopia—we followed the nonlinear MR approach of Staley and Burgess.^46^ First, the sample was stratified into seven refractive error quantiles (see below), with quantile 1 being comprised of the most myopic participants and quantile 7 the most hyperopic. Then, for each quantile, an MR analysis was performed exactly as described above. Heterogeneity across quantiles was assessed with Cochran's Q test (rma function in the metafor R package^47^). In practice, the seven refractive error quantiles were selected based not on refractive error per se but rather on the residual refractive error obtained after regressing refractive error on the polygenic score for refractive error and the covariates (age, age^2, age^3, gender, Northing, Easting, genotyping array, and the first 10 ancestry PCs). As described by Staley and Burgess,^46^ stratifying a sample into quantiles based on the “raw” exposure variable in the presence of confounders risks inducing a spurious association between the IV and the outcome, thereby violating the third core IV assumption. This was avoided by stratifying by the residual refractive error after conditioning on the IV.^46^

As a sensitivity analysis for violations of IV assumptions 2 and 3, we carried out the nonlinear MR analysis using the 129 SNPs as discrete instrumental variables rather than combining them into a single polygenic score. For the sensitivity analysis, regression coefficients describing the SNP-exposure and SNP-outcome relationships were calculated within each residual refractive error quantile. Information from the 129 instrumental variables within each quantile was then combined using inverse variance weighted (IVW) meta-analysis (rma function in the metafor R package). Heterogeneity across quantiles was assessed with Cochran's Q test. MR-Egger^48^ and weighted median-based MR^49^ were carried out, within each quantile, using the R package MendelianRandomization. The seven quantiles of residual refractive error for these sensitivity analysis were selected as described above, except that, instead of regressing refractive error on the polygenic score, refractive error was regressed on the single SNP being used as an IV, along with the covariates age, age^2, age^3, gender, Northing, Easting, genotyping array, and the first 10 ancestry PCs.

### Simulation Study

We carried out a simulation study to assess the statistical power of nonlinear MR to detect a causal effect of refractive error on years spent in education given the current analysis sample. In the simulation, a defined period of education was subtracted from the existing education level of participants with hyperopia exceeding a specific threshold level, followed by a test of whether the resulting nonlinear relationship between refractive error and education level could be detected using nonlinear MR. Power was calculated as the proportion of simulations in which the nonlinear relationship was detected. The details of the simulation were as follows. First, the observed outcome (EduYears) values in the study sample of 74,463 participants were permuted (i.e., randomly reassigned among participants without replacement), in order to break any existing link between refractive error and education level. Next, a specific period of education (ΔEduYears) of 0, 0.25, or 0.5 years was subtracted from the permuted EduYears value for those participants with hyperopia equal to or higher than a threshold level (*τ*Hyperopia) of +1.00 D, +2.00 D, or +3.00 D. This resulted in nine simulation conditions: 3 ΔEduYears conditions × 3 *τ*Hyperopia thresholds. Nonlinear MR analysis, with the polygenic score for refractive error as a single IV, was performed, as described above, to test for a causal effect of refractive error on education, followed by a test for heterogeneity across quantiles. The above steps were repeated 100 times per condition. Statistical power was calculated as the proportion of permutations in which a causal effect was detected for at least one of the hyperopia-dominated quantiles—that is, quantiles 5, 6, or 7 (*P* < 0.05 for test of causal effect in that quantile), conditional on the null hypothesis of no heterogeneity across quantiles being rejected (*P* < 0.05 for Cochran's Q test).

## Results

In the study sample of 74,463 UK Biobank participants, 52.7% were female, and the mean age was 57.9 years (median, 59.4 years; interquartile range [IQR], 12.2). On average, participants had a current (i.e., adult) refractive error of −0.23 D (median, +0.18 D; IQR, 2.3) and completed their full-time education when they were 18.2 years old (median, 18.0 years; IQR, 5.0). Approximately one-quarter of participants who, in adulthood, had hyperopia of less than +4.00 D reported wearing spectacles before the age of 16 years (Supplementary Table S2). This suggested that approximately three-quarters of participants with hyperopia of less than +4.00 D as adults did not wear spectacles during their school years, when they are likely to have been similarly or more hyperopic. In contrast, more than half of those with hyperopia above +4.00 D in adulthood reported wearing spectacles before the age of 16 years (Supplementary Table S2).

As reported previously for the UK Biobank cohort,^21^ an observational analysis using ordinary least squares (OLS) regression suggested that a more hyperopic refractive error was associated with fewer years spent in education. Each additional diopter of refractive error was associated with 9 weeks less time in full-time education (β = −0.18 yr/D; 95% CI, −0.19 to −0.17; *P* < 2e-16). The estimated effect was similar (β = −0.15 yr/D) after adjusting for a set of potential confounders (age, age^2, age^3, gender, Northing, Easting, genotyping array, and 10 ancestry PCs).

The polygenic score for refractive error explained 5.7% of the variance in refractive error in the study sample. Using the polygenic score as an instrumental variable, linear MR, which assumes that there is a linear relationship between refractive error and educational attainment, provided a causal effect estimate lower in magnitude than that obtained by OLS regression (β = −0.01 yr/D; 95% CI, −0.04 to +0.02; *P* = 0.52). The 95% CI of the linear MR causal effect estimate spanned zero, indicating minimal evidence for a non-zero causal effect. There was strong statistical support that the linear MR estimate was different from the OLS regression effect estimate (Wu–Hausman test statistic = 112.1; *P* < 2e-16), suggesting the influence of unmeasured confounders on the OLS estimate. Gauged using the R function IVpower, the linear MR analysis had 80% power to detect a causal effect of refractive error on years spent in education of −0.04 yr/D (2 weeks reduction in EduYears per diopter) or greater, assuming the exposure–outcome relationship was linear.

**Figure 2.**
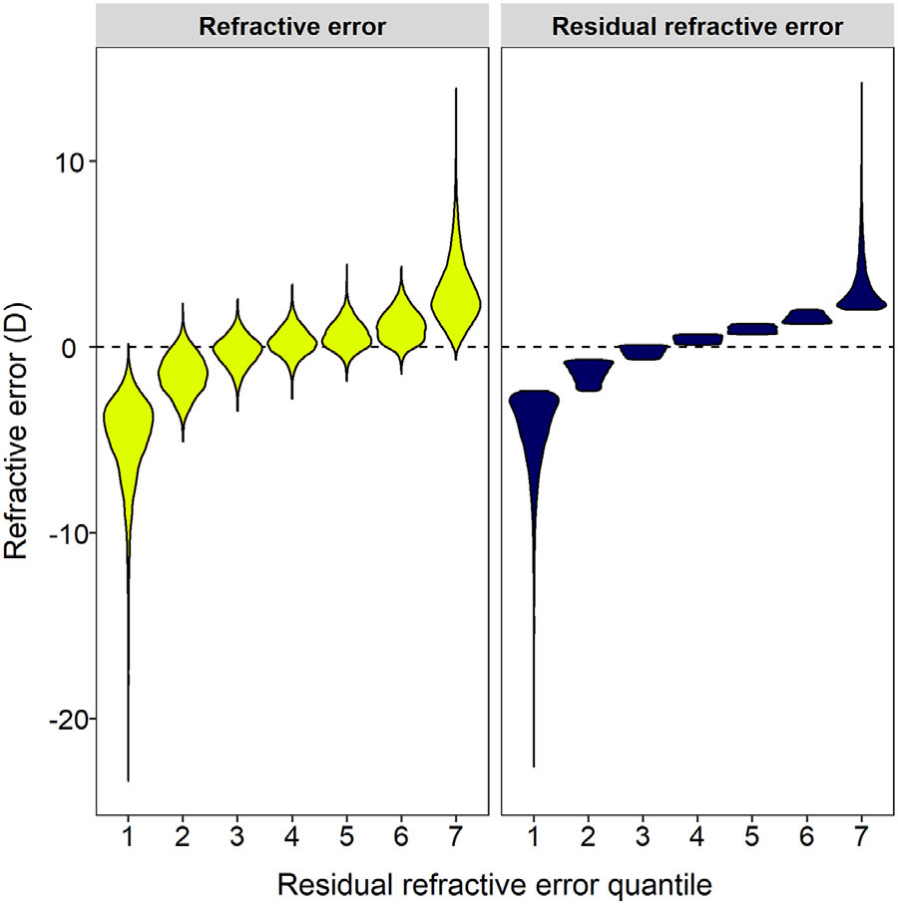
Distribution of refractive error and residual refractive error in quantiles of residual refractive error. Residual refractive error is the residual after regressing out the polygenic score for refractive error. Note that symbols are scaled to all have the same width.

**Figure 3.**
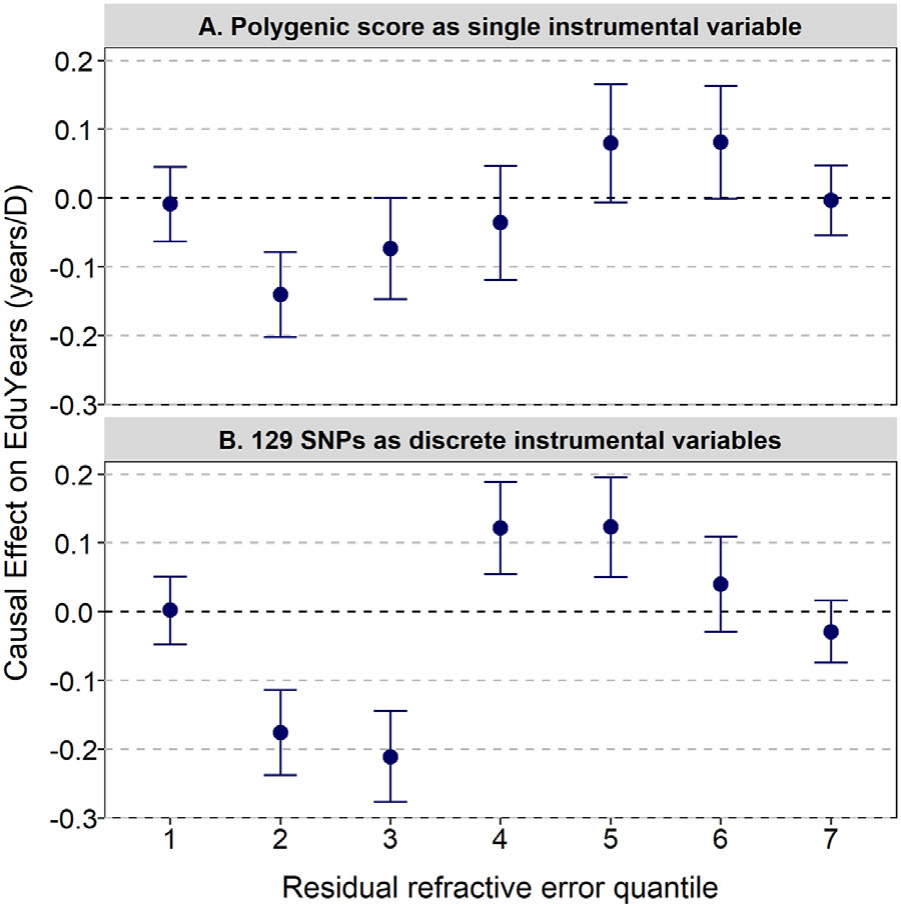
Estimated causal effect of refractive error on years spent in education (EduYears) obtained using nonlinear Mendelian randomization. *Error bars* are 95% CIs. The median residual refractive errors in quantiles 1 to 7 were −4.06 D, −1.33 D, −0.23 D, +0.41 D, +0.97 D, +1.59 D, and +2.81, respectively.

After stratifying the sample into seven quantiles of residual refractive error (i.e., refractive error adjusted to remove the effect associated with the polygenic score), quantiles 1 to 3 were enriched for participants with myopia, and quantiles 4 to 7 were enriched for participants with hyperopia (Fig. 2, Table 1). There were approximately 10,000 participants in each quantile. Nonlinear Mendelian randomization using the polygenic score for refractive error as an instrumental variable provided evidence that the causal effect of refractive error on educational attainment was indeed nonlinear, as indicated by a high level of heterogeneity across strata (*I*^2^ = 80.3%; Cochran's Q = 28.2; *P* = 8.8e-05). Quantile estimates of the causal effect of refractive error on EduYears ranged from −0.14 yr/D through to +0.08 yr/D (Fig. 3A, Table 2A). There was evidence supporting a non-zero causal effect for quantile 2 (β = −0.14 yr/D, 95% CI, −0.20 to −0.08; *P* = 8.7e-06), which corresponded to participants with a median refractive error of −1.45 D (range, −5.08 to +2.36 D), but not for the other quantiles. In summary, this analysis supported the hypothesis that there is a nonlinear causal relationship between refractive error and EduYears but did *not* support the hypothesis that hyperopia is a causal risk factor for lower educational attainment; indeed, the trend was in the opposite direction of a moderate level of hyperopia causing more time in education and a moderate level of myopia causing less time in education (Fig. 3A).

**Table 1.** Distribution of Refractive Error and Residual Refractive Error in Quantiles of Residual Refractive Error

			Refractive Error (D)			Residual Refractive Error (D)		
Quantile	Sample Size	EduYears (yr)	Minimum	Median	Maximum	Minimum	Median	Maximum
1	10,638	14.0	−23.35	−4.53	0.18	−22.59	−4.06	−2.36
2	10,638	13.5	−5.08	−1.45	2.36	−2.36	−1.33	−0.68
3	10,637	13.1	−3.41	−0.19	2.58	−0.68	−0.23	0.12
4	10,638	13.0	−2.76	0.26	3.36	0.12	0.41	0.69
5	10,637	13.0	−1.82	0.58	4.45	0.69	0.97	1.26
6	10,638	12.9	−1.43	1.09	4.35	1.26	1.59	2.02
7	10,637	12.6	−0.68	2.69	13.96	2.02	2.81	14.26

**Table 2.** Estimates of the Causal Effect of Refractive Error on EduYears in Nonlinear MR Analyses

Residual Refractive Error Quantile	*N*	*F*	Causal Effect (EduYears/D)	95% CI	*P*
A. Polygenic score as a single instrumental variable, external SNP weights					
1	10,638	1530	−0.009	−0.062 to 0.045	0.748
2	10,638	25,943	−0.140	−0.202 to −0.078	8.7e-06
3	10,637	81,311	−0.073	−0.147 to 0.001	0.051
4	10,638	127,134	−0.036	−0.119 to 0.047	0.392
5	10,637	120,171	0.080	−0.006 to 0.165	0.069
6	10,638	71,158	0.081	−0.001 to 0.163	0.054
7	10,637	4899	−0.004	−0.055 to 0.047	0.891
B. 129 SNPs as discrete instrumental variables, internal SNP weights, IVW meta-analysis					
1	10,638	—	0.002	−0.048 to 0.051	0.950
2	10,638	—	−0.176	−0.238 to −0.114	2.9e-08
3	10,637	—	−0.211	−0.277 to −0.144	6.0e-10
4	10,638	—	0.122	0.055 to 0.189	3.7e-04
5	10,637	—	0.123	0.050 to 0.195	9.7e-04
6	10,638	—	0.040	−0.029 to 0.109	0.255
7	10,637	—	−0.029	−0.074 to 0.016	0.213

Note that the *F* statistic will be different for each SNP in the IVW meta-analysis approach.

One of the assumptions of the nonlinear MR analysis is that the IV–exposure relationship has a constant magnitude across quantiles.^46^ In the current context, this is an assumption that the regression coefficient quantifying the association between the polygenic score and refractive error is constant across quantiles. Previous studies of myopia genetics suggest that this is not the case; polygenic scores and their constituent genetic variants are more strongly associated with refractive error in the extremes of the refractive error distribution than in the center.^30^^,^^50^ Specifically, genetic variants associated with refractive error have much larger effect sizes (beta coefficients) in individuals in the extreme myopic and hyperopic arms of the refractive error distribution than in individuals near the center of the distribution, an effect attributed to gene–gene or gene–environment interaction.^30^^,^^50^ We therefore carried out a sensitivity analysis that relaxed this assumption—namely, a nonlinear MR analysis that employed the 129 genetic variants associated with refractive error as separate instrumental variables rather than combined together as a polygenic score. Moreover, the effect size quantifying the association of each genetic variant with refractive error was calculated separately within each quantile, thereby accounting for differential genetic effect sizes across quantiles (but note that this approach required SNP weights to be calculated internally rather than using an external GWAS, which risks introducing bias from overfitting). The composite effect of all 129 IVs was calculated using inverse-variance weighted meta-analysis. The pattern of results for the sensitivity analysis was largely similar to that of the original nonlinear MR analysis (Fig. 3B, Table 2B). Once again, there was strong evidence of heterogeneity across strata (*I*^2^ = 94.6%; Cochran's Q = 91.1; *P* = 1.8e-17). However, in the sensitivity analysis, four quantiles—rather than just the single quantile observed in the original analysis—displayed evidence of a causal relationship between refractive error and educational attainment. Specifically, in quantiles 2 and 3, which were enriched for participants with myopia, refractive error was causally associated with fewer years in education (β = −0.18 yr/D and −0.21 yr/D, respectively), whereas in quantiles 4 and 5, which were enriched with emmetropes and low hyperopes, refractive error was causally associated with more years in education (β = +0.12 yr/D for both quantiles). In summary, this sensitivity analysis also provided support for a nonlinear causal relationship between refractive error and education, but not for the hypothesis that hyperopia causes fewer years in education. A further sensitivity analysis was performed to evaluate if the MR causal estimate in each quantile was strongly influenced by any single genetic variant. This “leave-one-out” sensitivity analysis did not suggest that any individual genetic variants had a major impact on the results (Supplementary Fig. S3).

**Figure 4.**
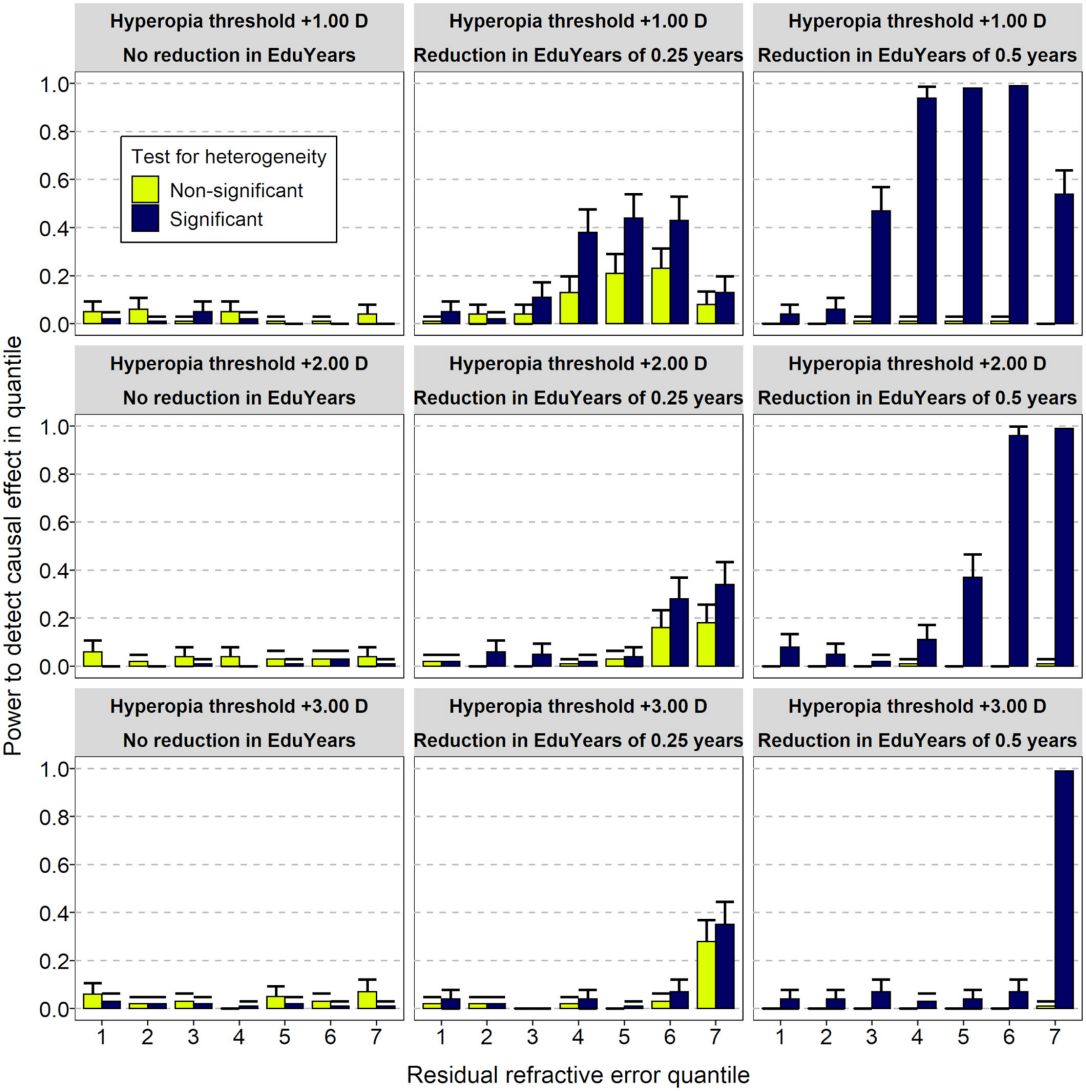
Simulation to assess power of nonlinear MR to detect a reduction in years spent in education (EduYears) in participants with hyperopia. Simulations were run in which a defined period of education (0, 0.25 or 0.5 years) was subtracted from participants with hyperopia above a threshold level (+1.00, +2.00, or +3.00 D). Power was calculated as the proportion of replicates in which nonlinear MR analysis identified a non-zero causal effect in a quantile, stratified by whether heterogeneity across quantiles was detected (i.e., Cochran's Q test, *P* < 0.05).

**Figure 5.**
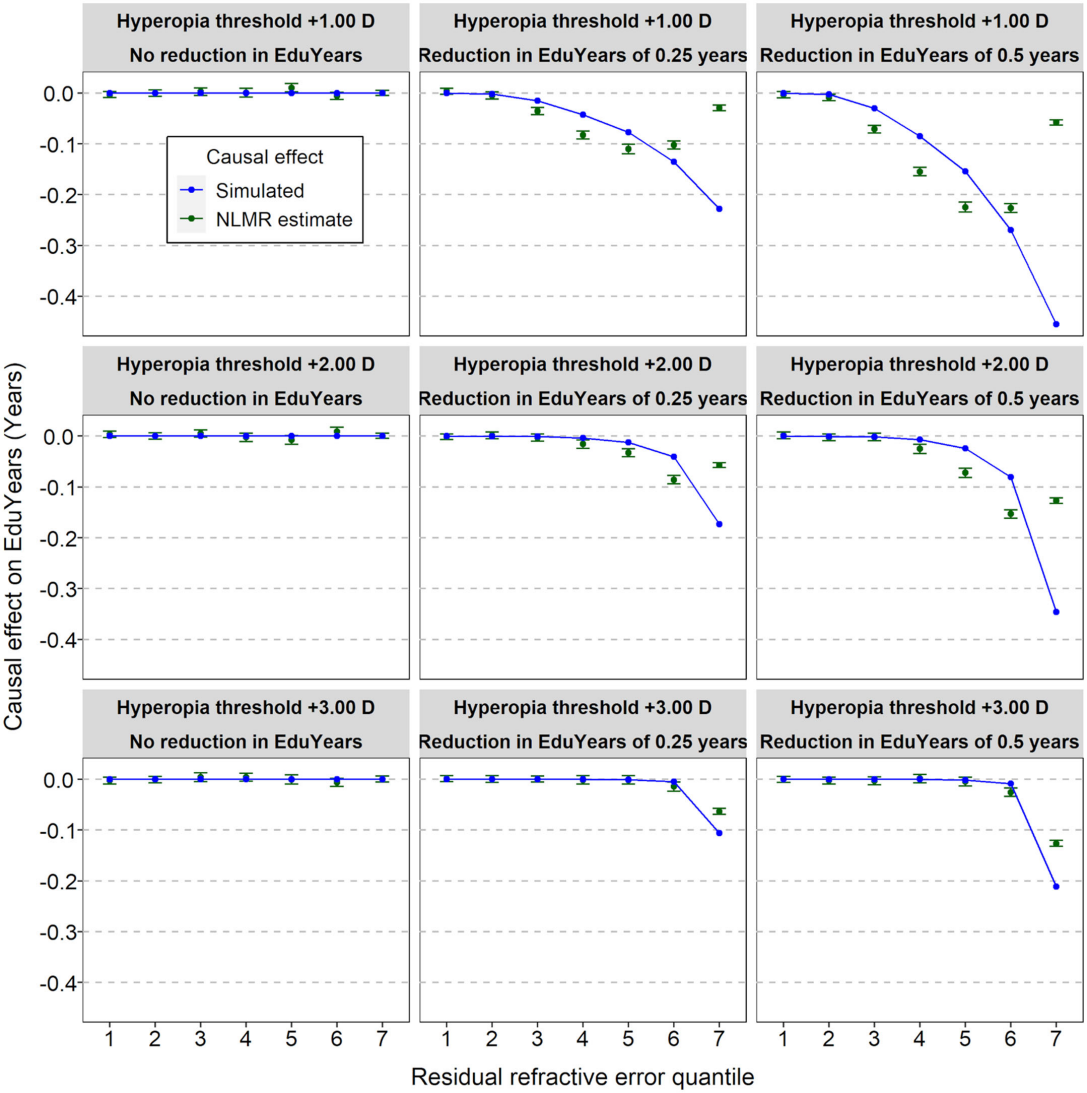
Simulation to assess power of nonlinear MR to detect a reduction in years spent in education (EduYears) in participants with hyperopia. Simulations were run in which a defined period of education (0, 0.25, or 0.5 years) was subtracted from participants with hyperopia above a threshold level (+1.00, +2.00, or +3.00 D). The blue curve indicates the simulated causal effect on EduYears. The green points show the causal effect estimated using nonlinear MR (*error bars* indicate 95% CIs of 100 replicate simulations). NLMR, nonlinear Mendelian randomization.

**Figure 6.**
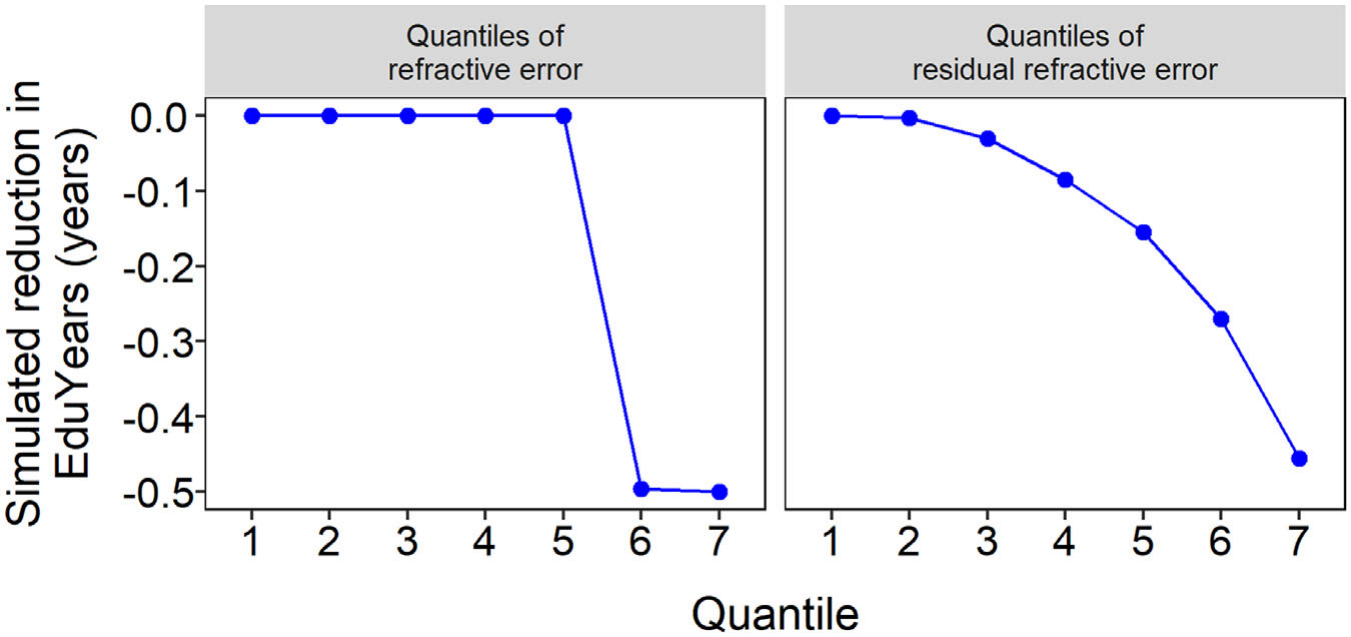
Comparison of patterns of simulated reduction in EduYears in quantiles of refractive error and quantiles of residual refractive error. Results are presented for a simulated deficit in EduYears of −0.5 years in all participants with hyperopia of +1.00 D or more. When this simulated reduction in EduYears was considered in terms of refractive error quantiles, the pattern differed for “raw” (non-residual) refractive error quantiles compared with residual refractive error quantiles. Note the abrupt reduction in EduYears in the two highest quantiles of “raw” refractive error, but the only gradual reduction in EduYears across increasing quantiles of residual refractive error.

Simulations were carried out to assess the statistical power of nonlinear MR analysis in the UK Biobank sample to detect a causal effect of hyperopia on educational attainment. A deficit in EduYears (ΔEduYears) of 0, 3, or 6 months was simulated in participants with hyperopia at or above a threshold level (*τ*Hyperopia) of +1.00, +2.00, or +3.00 D. The results of the simulation analyses are presented in Figures 4 and 5. When the simulated deficit in EduYears was zero (ΔEduYears *=* 0), the type I error rate was close to the nominal level of 0.05. When the deficit in EduYears was simulated to be 3 or 6 months, there was a trend of increasing power when the deficit in years spent in education was larger and when the threshold level of hyperopia at which a causal effect arose was lower (e.g., power was greater when *τ*Hyperopia = +2.00 D than when *τ*Hyperopia = +3.00 D). However, the power to detect a non-zero causal effect only exceeded 0.8 when ΔEduYears was simulated to be 6 months (Fig. 4). Furthermore, as evident in Figure 5 (blue curves), the simulated level of EduYears did not reduce abruptly at the *τ*Hyperopia threshold when considering residual refractive error quantiles. As shown in Figure 6, this pattern occurred despite the simulated boundary in “raw” (non-residual) refractive error actually being an abrupt transition; that is, ΔEduYears was zero below the *τ*Hyperopia threshold and non-zero above the *τ*Hyperopia threshold. This phenomenon meant that nonlinear MR causal estimates from the simulations did not show a sharp boundary at the simulated *τ*Hyperopia threshold. Thus, the simulations suggested it would be challenging to infer the precise refractive error threshold at which any deficit in EduYears began, based on a nonlinear MR analysis.

## Discussion

The previous MR study of Mountjoy et al.^21^ suggested that, on average, refractive error has no causal effect on years spent in education. The current findings build on this by suggesting that refractive error has a nonlinear influence on EduYears (*P* = 8.8e-05), being zero on average, but with a general trend of less myopia in those with moderate myopic refractive errors causing a few weeks less time to be spent in education and more hyperopia in those with moderate hyperopic refractive errors causing a few weeks more time to be spent in education (Fig. 3). These findings are counterintuitive. However, we speculate that the small deficit in educational attainment in the quantiles enriched for participants with low to moderate myopia may have been caused by some of these participants having uncorrected myopia during their school years. Uncorrected myopia is common worldwide, especially in groups who experience barriers to access eye care, due to either socioeconomic factors or lack of local services.^51^^–^^56^ Children with myopia who do not have spectacles, have spectacles whose spectacle prescription is outdated or inaccurate, or who choose not to wear their spectacles for school, will experience blurred distance vision. This may hinder their educational attainment.^57^ Notably, the causal trend from the MR analysis was very different from the trend observed in an OLS regression analysis, in which hyperopia was associated with less time in education and myopia was associated with more time in education. A potential reason for the conflicting results of the observational analysis results and the nonlinear MR results is a causal effect of education on refractive error.^20^^,^^21^^,^^31^ In other words, the observational association between refractive error and education may be dominated by the pathway education → refractive error, whereas the nonlinear refractive error → education pathway may have much less influence.

The major strength of this work is that MR provides a strategy for studying the relationship between hyperopia and years spent in education that is subject to different limitations and sources of bias compared to those present in observational clinical studies, which have been the mainstay for investigating the relationship previously. Thus, the new findings provide largely independent evidence to add to what was known before. However, it is important to interpret the nonlinear MR results cautiously. Compared with the linear MR analysis, which had high statistical power, our simulation study suggested that the nonlinear MR approach had limited power, except when the hyperopia-induced deficit in EduYears was approximately 6 months per diopter or more. If children with hyperopia truly suffer a deficit in education of less than 6 months per diopter, then the current study may have failed to detect this effect due to lack of statistical power. Furthermore, the validity of the nonlinear MR approach is dependent on a series of assumptions, some of which cannot be tested directly. These assumptions are as follows.

Assumption A (core instrumental variable assumption 1) assumes that the polygenic score used as an IV is associated with refractive error in childhood. The polygenic score and its constituent SNPs were derived from a large-scale GWAS meta-analysis for refractive error in adults; however, as shown in Supplementary Table S3, the polygenic score was also predictive of refractive error across childhood, justifying this assumption.

Assumptions B and C (core instrumental variable assumptions 2 and 3) assume that, to be a valid IV, the polygenic score and its constituent SNPs must act independently of unmeasured confounding factors. Also, the association between the polygenic score and EduYears should be mediated solely through refractive error. A confounding bias plot^45^ (Supplementary Fig. S5) suggested that there was relatively less bias across a range of measured potential confounders in the IV analysis compared to an observational (OLS) analysis, except for greater relative bias from ancestry PC7. These results are consistent with those obtained using a closely related polygenic score for refractive error by Mountjoy et al.^21^ Furthermore, the nonlinear MR results were similar with and without adjustment for the measured potential confounders, age, age^2, age^3, gender, Northing, Easting, genotyping array, and the first 10 ancestry genetic principal components (Supplementary Fig. S4). Note that, although we included the Townsend deprivation index (TDI) in our confounding bias analysis, the TDI was not included as a covariate in any of our other analyses. The rationale for this was that information on TDI was only available for the participants at their current age of 40 to 70 years old; hence, this measure of socioeconomic status may have been, in part, a reflection of the participants’ educational attainment (i.e., education → TDI rather than TDI → education). Ideally, the TDI of participants during childhood would have been included as a covariate in our analyses, as this may have better captured the causal effects of socioeconomic status on educational attainment.^58^ The MR sensitivity analysis methods, MR-Egger and weighted median-based MR, can provide valid causal estimates when a proportion of genetic variants are invalid IVs.^48^^,^^49^ These additional sensitivity analyses produced causal effect estimates comparable to those for the original MR analysis (Supplementary Tables S4 and S5) and led to the same conclusions as the main analysis. Thus, although assumptions 2 and 3 cannot be proven to be valid,^44^ the available evidence was consistent with their validity.

Assumption D assumes that hyperopia present in adulthood would have been present in childhood. There is a large body of evidence that, on average, children's refractive errors tend to progress toward myopia rather than hyperopia during the school years.^59^^–^^61^ These studies report that it is common for individual children with hyperopia to become emmetropic or myopic as they get older, but that it is rare for a child who is not hyperopic in childhood to become hyperopic by the time they reach middle age.

Assumption E assumes that years spent in full-time education is a valid proxy for educational attainment. Years spent in education (EduYears) has been used as a measure of educational attainment in several GWASs,^36^^,^^62^^,^^63^ and this trait varied widely in the current study sample; approximately 40% of participants reported 11 years or fewer of full-time education and another 40% reported 16 years or more of full-time education. In support of the validity of Assumption E, it has been reported that EduYears and the binary variable “college completion (yes/no)” have a very high genetic correlation.^36^ Nevertheless, important limitations of the use of EduYears as a measure of educational attainment are that it reflects years in education and not actual academic achievement, it does not take account of different types of educational activity or early life experiences, and it does not take account of individuals who have breaks in education for any reason.

Assumption F assumes that prescribing spectacles to some children with hyperopia does not impact the overall relationship between refractive error and education. If hyperopia lowers educational attainment via a mechanism relating to visual function, then children with hyperopia who receive spectacles should have a lesser deficit in their educational attainment compared with children who do not receive spectacles. There is mixed evidence from the literature regarding whether correction of hyperopia disrupts emmetropization.^64^^–^^66^ For the current sample, approximately 25% of individuals with moderate hyperopia and 60% of those with high hyperopia reported wearing spectacles during their school years (Supplementary Table S2). Importantly, therefore, our MR analysis may have missed any adverse effects on educational attainment that would have been present if none of the children had received spectacles. The factors influencing whether a child with hyperopia is prescribed (and wears) spectacles include socioeconomic status. For this reason, we did not include age of onset of spectacle wear (AOSW) as a covariate in our analyses, as this may have introduced collider bias^67^; that is, refractive error and socioeconomic status would both be causes of AOSW. Given that approximately 60% of participants with hyperopia above +4.00 D reported having spectacles during their school years, our nonlinear MR analyses had limited scope to identify potential adverse effects on educational attainment for children with this level of refractive error. (We speculate that many of the participants who had myopia during their childhood may also have worn spectacles for schoolwork, which may have biased the nonlinear MR causal effect estimate toward the null for quantiles enriched for participants with myopia. We also note that fewer children with hyperopia growing up in the United Kingdom from the 1950s to the 1970s may have received spectacles than would be the case in recent decades.^68^) Hypothetically, in an ideal MR study designed to address the relationship between hyperopia and educational attainment, none of the children in the study would be prescribed spectacles. This would allow the full extent of any adverse consequences of hyperopia to become manifest; however, such a idealized study would be unethical to carry out on the scale necessary for an MR analysis. (By contrast, a smaller scale RCT in which children with hyperopia were randomized to receive or not receive spectacles could potentially be designed in a way that addressed ethical concerns).

In summary, a Mendelian randomization analysis provided strong evidence for a nonlinear causal relationship between refractive error and years spent in education. There was suggestive evidence that a moderate level of myopia caused a small reduction in years of education (approximately 7 weeks lower duration of full time education per diopter) and therefore was likely to be dominated by the larger causal effect in the opposite direction (education causing myopia), known to exist from previous work. Our principal finding was that there was minimal evidence to support a major deficit in educational attainment caused by a moderate level of hyperopia in childhood. However, this result is subject to two important caveats. First, approximately 20% of participants who were likely to have been moderately hyperopic in childhood reported having spectacles before they were 16 years old. In these children, wearing spectacles may have negated any adverse effects of moderate hyperopia on their education, such that a truly causal relationship may not have been revealed by our analysis. Second, the current study had limited statistical power, which meant that only large deficits in educational attainment (a deficit of 6 months or more per diopter of hyperopia) could be ruled out. This work further highlights the need for randomized controlled trials to investigate the relationship between hyperopia and educational attainment.

## Supplementary Material

Supplement 1
