# Supplementary material for: Hyperopia Is Not Causally Associated With a Major Deficit in Educational Attainment
Source: Transl Vis Sci Technol. 2021 Oct 28;10(12):34. doi: 10.1167/tvst.10.12.34 (PMC8556559; doi:10.1167/tvst.10.12.34)
Supplement: Supplement 1 [file tvst-10-12-34_s001.pdf]

Hyperopia is not causally associated with a major deficit in educational attainment

Supplementary Information

Item	Title	Page
Supplementary Table S1.	Genetic variants associated with refractive error used as instrumental variables.	2
Supplementary Table S2.	Self-reported age-of-onset of spectacle wear (AOSW) stratified by refractive error in adulthood.	6
Supplementary Table S3.	Childhood refractive error variance explained (R^2) by the polygenic score.	7
Supplementary Table S4.	MR-Egger sensitivity analysis.	8
Supplementary Table S5.	Weighted median-based MR sensitivity analysis.	9
Supplementary Figure S1.	Flow chart illustrating participant selection and exclusion steps	10
Supplementary Figure S2.	Comparison of externally-derived squared Z-scores and internally-derived regression (beta) coefficients as potential SNP weights.	11
Supplementary Figure S3.	Estimated causal effect of refractive error on years spent in education (EduYears) obtained using non-linear Mendelian randomization in a leave-one-out analysis.	12
Supplementary Figure S4.	Estimated causal effect of refractive error on years spent in education (EduYears) obtained using non-linear Mendelian randomization without adjustment for covariates.	13
Supplementary Figure S5.	Confounding bias plot.	14

Supplementary Table S1. Genetic variants associated with refractive error used as instrumental variables. These variants were reported by Tedja et al.[1] in a CREAM Consortium study. For CREAM variants not available in UK Biobank, a surrogate variant in high linkage disequilibrium was selected. Squared Z-scores from Tedja et al.[1] were used as weighting factors to create the polygenic score. Abbreviations: SNP, single nucleotide polymorphism; CHR, chromosome, POS, genomic position for build GRCh37; A1, first allele; A2, second allele; A1F, frequency of allele A1 in European population; SNP_EA, single nucleotide polymorphism and effect allele; Z-score^2, squared Z-score weighting for each copy of the effect allele (EA).

CREAM Consortium variant						UK Biobank variant	
**SNP**	**CHR**	**POS**	**A1**	**A2**	**A1F**	**SNP_EA**	**Z-score^2**
rs11210537	1	42345723	A	G	0.3393	rs11210537_A	33.9190
rs11589487	1	61342229	A	G	0.4451	rs11589487_A	44.4222
rs1237670	1	113418415	A	G	0.7753	rs1237670_G	-33.8142
rs11802995	1	158053024	A	C	0.7592	rs11802995_C	-38.4772
rs1556867	1	164213686	T	C	0.2641	rs1556867_T	-77.5456
rs2225986	1	200311910	A	T	0.3812	rs2225986_A	-63.4094
rs1858001	1	207488004	C	G	0.6761	rs1858001_G	-52.9402
rs2745953	1	208062973	A	T	0.7128	rs2745953_T	-33.1661
rs11118367	1	219790221	T	C	0.4824	rs11118367_T	-53.1149
rs6753137	2	301051	T	C	0.4556	rs6753137_T	-40.8321
rs28658452	2	16234068	A	G	0.9147	rs28658452_G	30.1401
rs17032696	2	45137870	A	C	0.7718	rs17032696_C	33.2468
rs41393947	2	56011517	A	G	0.132	rs41393947_A	-41.2164
rs10187371	2	145226370	T	C	0.1862	rs10187371_T	-43.0467
rs56075542	2	146882415	T	G	0.5523	rs56075542_G	80.8920
rs297593	2	157363743	T	C	0.2856	rs297593_T	-61.0899
rs17428076	2	172851936	C	G	0.768	rs17428076_G	66.9615
rs6433704	2	178847912	T	G	0.6032	rs6433704_G	-32.7527
rs2573232	2	233300046	T	C	0.9001	rs2573232_C	53.5531
rs2573210	2	233385025	A	G	0.8066	rs2573210_G	-155.2770
rs9681162	3	8194734	T	C	0.6799	rs9681162_C	-44.8766
rs1454776	3	16009044	T	G	0.4939	rs1454776_G	-44.2225
rs4260345	3	24256698	T	C	0.6208	rs4260345_C	-32.0582
rs4687586	3	53837971	C	G	0.6905	rs4687586_G	42.8632
rs7624084	3	141093285	T	C	0.5676	rs7624084_C	77.6337
rs4894529	3	171959684	A	G	0.4833	rs4894529_A	31.8773
rs7662551	4	80537638	A	G	0.7232	rs7662551_G	-72.7609

rs7747	4	80827062	T	C	0.2022	rs7747_T	49.4349
rs7692381	4	81903049	A	G	0.7625	rs7667446_C	-88.3224
rs74764079	4	81952637	A	T	0.0227	rs74764079_A	-73.8225
rs2166181	4	82422327	A	G	0.519	rs2166181_G	-44.1162
rs11952819	5	71780033	T	C	0.2763	rs11952819_T	-33.5820
rs7737179	5	87795525	A	G	0.2542	rs7737179_A	-30.5477
rs7449443	5	174720893	T	G	0.5968	rs7449443_G	30.3601
rs10458138	6	2429743	A	G	0.2377	rs10458138_A	-36.5904
rs9295499	6	21160689	A	C	0.3158	rs9295499_A	30.5698
rs1928175	6	22079485	A	G	0.5508	rs1928175_G	54.4792
rs1150687	6	28162469	T	C	0.6192	rs1150687_C	-46.0227
rs9395623	6	50757699	A	T	0.3149	rs9395623_A	52.5625
rs7744813	6	73643289	A	C	0.5905	rs7744813_C	211.848
rs12526735	6	73648822	A	T	0.5034	rs12526735_A	52.43208
rs1064583	6	116446576	A	G	0.6162	rs1064583_G	-42.5495
rs12193446	6	129820038	A	G	0.9063	rs12193446_G	377.5638
rs9388766	6	130354855	T	C	0.298	rs9388766_T	38.8628
rs1358684	7	86103402	T	C	0.7086	rs1358684_C	-30.3821
rs73730144	7	158862984	A	C	0.9783	rs73730144_C	-32.6041
rs2116093	8	10613299	C	G	0.4303	rs2116093_C	30.5477
rs1532278	8	27466315	T	C	0.3837	rs1532278_T	-32.3533
rs7829127	8	40726394	A	G	0.7917	rs7829127_G	119.0499
rs284816	8	53362145	A	G	0.1632	rs284818_T	-52.0129
rs3110134	8	60097984	A	G	0.3231	rs3110134_A	37.5033
rs72655575	8	60556509	A	C	0.2013	rs72655575_A	47.1557
rs28891973	8	71420067	A	G	0.4246	rs2622646_A	39.3129
8:121622778:D	8	121622778	G	GT	0.5647	rs55885222_A	-34.8100
rs10511652	9	18362865	A	G	0.416	rs10511652_A	54.09603
rs11145465	9	71766593	A	C	0.2122	rs11145465_A	-91.1261
rs7042950	9	77149837	A	G	0.7323	rs7042950_G	-46.1992
rs10760673	9	101878622	A	G	0.2274	rs10760673_A	-35.8322
rs10122788	9	129206832	A	G	0.5603	rs10122788_G	-30.1511
rs11101263	10	49414181	T	C	0.2582	rs11101263_T	-53.7142
rs1649068	10	60304864	A	C	0.4747	rs1649068_A	-89.0947
rs9416017	10	74100279	T	C	0.4034	rs9416017_T	-31.9790
rs4237284	10	77814847	A	G	0.6024	rs4237285_T	29.98658
rs7895108	10	79061458	T	G	0.3513	rs7895108_T	-78.6060
rs10887262	10	86009171	T	C	0.7075	rs10887262_C	-50.6659
rs11202736	10	90142203	A	T	0.7168	rs11202736_T	47.8726
rs17382981	10	94953258	T	C	0.4172	rs17382981_T	-39.8666
rs807037	10	102824349	C	G	0.6532	rs807037_G	29.87716
rs511217	11	30029948	A	T	0.738	rs511217_T	46.14485
rs7941828	11	30430331	T	C	0.3396	rs7941828_T	30.06329
rs11602008	11	40149305	A	T	0.822	rs11602008_T	-195.3840
rs7107014	11	43307811	A	C	0.4973	rs7107014_A	29.80068
rs2155413	11	84634790	A	C	0.4823	rs2155413_A	-60.1400

rs1954761	11	105596885	T	C	0.371	rs1954761_T	-70.5096
rs7122817	11	117657679	A	G	0.5072	rs7122817_A	56.4602
11:128787963:D	11	128787963	A	AC	0.6903	rs7933504_G	-30.7470
rs1790165	11	131928971	A	C	0.4113	rs1790165_A	46.97732
rs5442	12	6954864	A	G	0.0683	rs5442_A	-61.0742
rs7968679	12	9313304	A	G	0.7004	rs7968679_G	-48.3025
rs4764038	12	14062637	T	G	0.2543	rs4764038_T	-38.3904
rs7971334	12	20720707	T	G	0.3065	rs7971334_T	-32.7642
rs117735470	12	22592653	A	G	0.0894	rs117735470_A	-36.8449
rs10880855	12	46144855	T	C	0.5068	rs10880855_T	-60.4973
rs3138137	12	56116981	A	C	0.4646	rs3138137_A	113.7636
rs11178469	12	71275137	T	C	0.7524	rs11178469_C	54.80441
rs7337610	13	28962666	T	C	0.391	rs7337610_T	-37.7487
rs1359543	13	50159165	A	G	0.5794	rs1359543_G	39.7026
rs9547035	13	85573496	T	G	0.716	rs9547035_G	-32.342
rs9516194	13	93859498	A	G	0.4901	rs9516194_A	39.00003
rs9517964	13	100717833	T	C	0.589	rs9517964_C	-70.9469
rs837323	13	101175664	T	C	0.5115	rs837323_T	39.91712
rs12883788	14	33303540	T	C	0.425	rs12883788_T	38.98754
rs36024104	14	42294993	A	G	0.8227	rs36024104_G	-82.6281
rs2855530	14	54421917	C	G	0.5067	rs2855530_C	-73.5306
rs2143964	14	54726800	C	G	0.7266	rs2143964_G	-55.1009
rs2753462	14	60850703	C	G	0.2955	rs1313240_C	-42.172
rs17125093	14	89428948	A	G	0.2163	rs17125093_A	-38.8378
rs11160044	14	92582655	A	T	0.2086	rs56014528_T	42.65396
rs35337422	14	104407243	A	C	0.8552	rs35337422_C	-40.2844
rs524952	15	35005886	A	T	0.4748	rs524952_A	-291.556
rs34539187	15	48756536	C	G	0.88	rs34539187_G	33.73286
rs12898755	15	63574641	A	G	0.2453	rs12898755_A	56.74609
rs6495367	15	79375347	A	G	0.4075	rs6495367_A	-104.081
rs1969091	15	82326775	A	C	0.2921	rs1969091_A	40.51323
rs28471081	16	7414383	A	G	0.7828	rs28471081_G	48.23303
rs10500355	16	7459347	A	T	0.3543	rs10500355_A	-188.568
rs56055503	16	80532694	A	G	0.7513	rs56055503_G	45.14496
rs8075280	17	7434819	A	T	0.4211	rs8075280_A	33.10852
rs2908972	17	11407259	A	T	0.4146	rs2908972_A	-123.766
rs80253120	17	14138507	T	C	0.6259	rs115152181_A	-48.5252
rs62070229	17	31227593	A	G	0.8065	rs62070229_G	-73.5821
rs4795364	17	37576546	A	G	0.744	rs4795364_G	-30.603
rs11654644	17	47263475	T	C	0.2101	rs11654644_T	-39.8919
rs12451582	17	54734643	A	G	0.3687	rs12451582_A	49.2804
17:56619441:I	17	56619441	CA	C	0.2504	rs8073754_T	-35.4501
rs4793501	17	68718734	T	C	0.5748	rs4793501_C	52.01294
rs7207217	17	69528353	A	G	0.3965	rs7207217_A	-30.9581
rs6420484	17	79612397	A	G	0.3554	rs6420484_A	-35.3192
rs10853531	18	42824449	A	G	0.1996	rs10853531_A	47.36192

rs12965607	18	47391025	T	G	0.8573	rs12965607_G	-50.0273
rs4808962	19	19579557	A	G	0.8296	rs4808962_G	-36.6751
rs235770	20	6761765	T	C	0.3717	rs235770_T	-35.1175
rs1555075	20	32610401	T	C	0.3145	rs1555075_T	38.62623
rs2229742	21	16339172	C	G	0.1047	rs2229742_C	-37.4666
rs11088317	21	16574122	T	C	0.2871	rs11088317_T	-47.541
rs9680365	21	30928732	A	G	0.04	rs9680365_A	-33.074
rs7275394	21	32776107	A	G	0.0245	rs72826094_T	-30.5809
rs2150458	21	47377296	A	G	0.4546	rs2150458_A	59.83023
rs9606967	22	32899516	C	G	0.2061	rs9606967_C	-34.41

Supplementary Table S2. Self-reported age-of-onset of spectacle wear (AOSW) stratified by refractive error in adulthood. Note that 8,445 participants in the full sample of 74,463 did not wear spectacles (and therefore did not have an AOSW*).

Refractive error category in adulthood	Number of participants (with known AOSW)	Number of participants (total)	Self-reported age-of-onset of spectacle wear (years)			Percentage with self-reported age-of-onset of spectacle wear before specified age (%)				
			Mean	Median	Range	5 yrs	7 yrs	10 yrs	13 yrs	16 yrs
-3.01 and below	9,703	9,821	13.3	12	1 – 65	1.4	6.7	25.0	53.4	73.6
-3.00 to -2.01	3,915	4,030	18.9	17	1 – 65	0.7	2.5	8.1	23.6	42.4
-2.00 to -1.01	5,571	6,023	24.8	21	1 – 68	0.5	1.7	4.7	13.7	26.0
-1.00 to -0.01	10,176	13,496	36.2	40	1 – 67	0.5	1.3	2.8	5.9	9.9
0.00 to +0.99	16,457	19,901	42.5	45	1 – 69	0.5	1.3	2.3	3.9	5.5
+1.00 to +1.99	10,835	11,425	41.6	45	1 – 69	1.3	2.7	4.2	6.1	7.8
+2.00 to +2.99	4,816	5,047	37.4	42	1 – 65	2.7	5.5	8.6	12.1	14.2
+3.00 to +3.99	2,156	2,274	29.7	35	1 – 64	6.3	12.0	17.7	23.0	26.3
+4.00 and above	2,389	2,446	16.0	10	1 – 60	21.0	36.2	47.0	56.0	60.9

*Percentage with self-reported AOSW before a specified age was calculated only for the subsample with a known AOSW.

Supplementary Table S3. Childhood refractive error variance explained (*R^2*) by the polygenic score. Refractive error at different ages of childhood was regressed against the polygenic score (derived using the 129 genetic variants listed in Supplementary Table S1 and weighted by the squared Z-score from Tedja et al.[1]). Details of the childhood refractive error study sample and methods have been reported previously.[2] The p-value is from a test of the null hypothesis that the polygenic score is not associated with refractive error.

Age (years)	Sample size	R-squared (%)	*P*
7	5,564	1.07	6.58e-15
10	5,291	1.82	3.71e-23
11	4,832	2.3	1.75e-26
12	4,839	2.24	8.22e-26
15	3,687	2.98	2.62e-26

Supplementary Table S4. MR-Egger sensitivity analysis. The intercept term from an MR-Egger analysis provides an indication of directional horizontal pleiotropy. The causal effect estimate is valid in the presence of directional pleiotropy. Evidence for directional pleiotropy was seen in quantiles 5 and 7, although the magnitude was small. The pattern of MR-Egger causal effect estimates was similar to the original MR analysis across all quantiles.

Quantile	MR-Egger Intercept			MR-Egger Causal Effect		
	Estimate	95% CI	*P*	Estimate	95% CI	*P*
1	0.009	(-0.003 to 0.022)	0.124	-0.061	(-0.155 to 0.032)	0.200
2	-0.002	(-0.017 to 0.014)	0.841	-0.163	(-0.301 to -0.025)	0.020
3	-0.008	(-0.023 to 0.006)	0.267	-0.137	(-0.283 to 0.009)	0.066
4	0.011	(-0.003 to 0.026)	0.131	0.019	(-0.131 to 0.169)	0.807
5	0.022	(0.007 to 0.037)	0.004	-0.090	(-0.250 to 0.071)	0.275
6	-0.006	(-0.021 to 0.009)	0.409	0.099	(-0.056 to 0.253)	0.212
7	-0.018	(-0.032 to -0.004)	0.010	0.080	(-0.014 to 0.174)	0.095

Supplementary Table S5. Weighted median-based MR sensitivity analysis. The causal effect estimate from a median-based MR analysis is valid even if up to half of the SNPs are invalid IVs. The pattern of Median-based MR causal effect estimates was similar to the original MR analysis across quantiles.

Quantile	Weighted Median-based MR Causal Effect Estimate	95% CI	*P*
1	-0.037	(-0.111 to 0.037)	0.326
2	-0.194	(-0.291 to -0.097)	<0.001
3	-0.125	(-0.233 to -0.018)	0.022
4	0.117	(0.013 to 0.221)	0.028
5	0.088	(-0.027 to 0.204)	0.133
6	0.038	(-0.069 to 0.144)	0.488
7	0.001	(-0.071 to 0.073)	0.980

Supplementary Figure S1. Flow chart illustrating participant selection and exclusion steps.

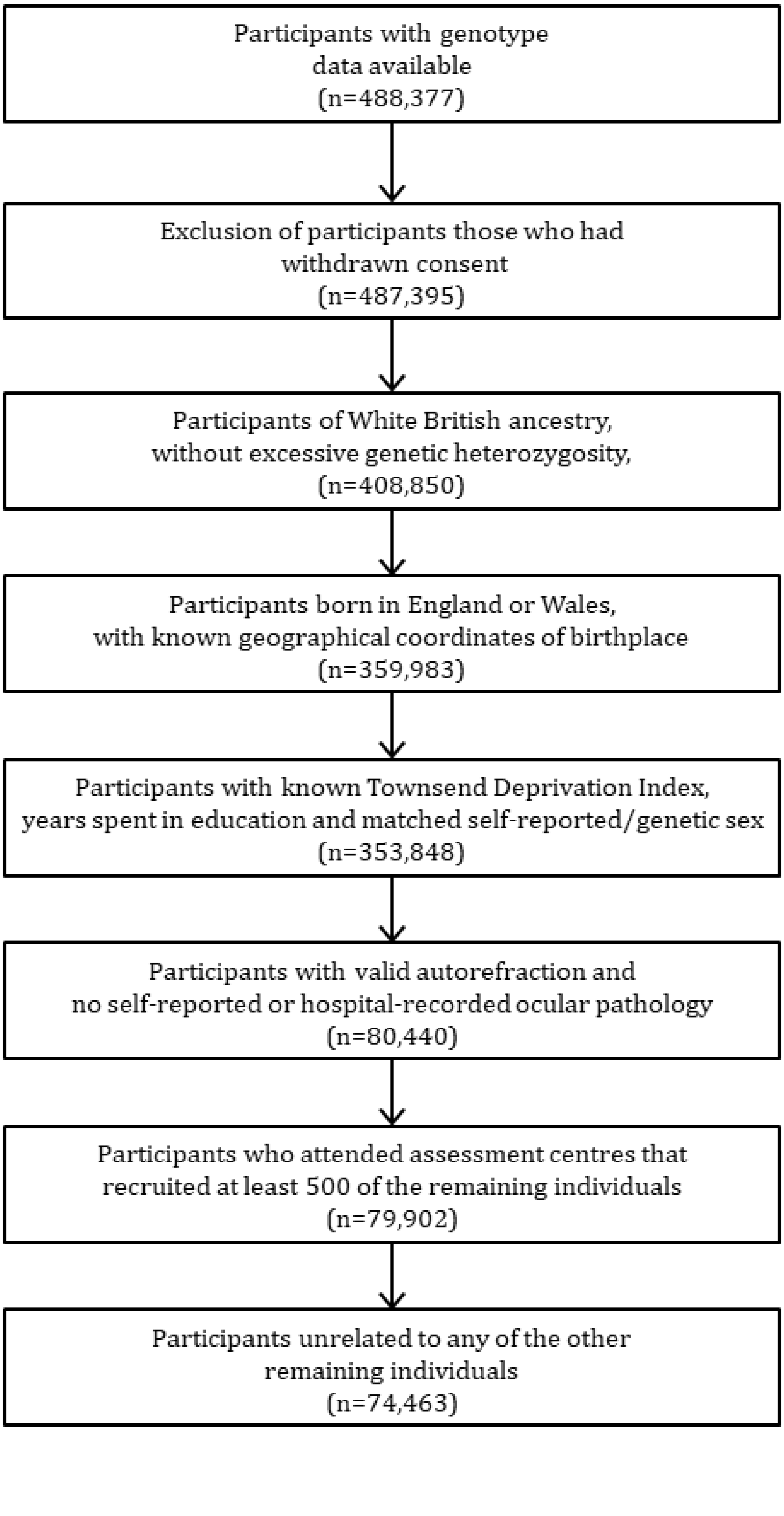

Supplementary Figure S2. Comparison of externally-derived squared Z-scores and internally-derived regression (beta) coefficients as potential SNP weights. Z-scores were those reported by Tedja et al.[1] from an analysis of approximately 160,000 participants from CREAM and 23andMe Inc. that did not include UK Biobank participants (Supplementary Table S1). Regression (beta) coefficients were calculated for the 74,463 UK Biobank participants in the non-linear MR analysis sample. Note the relationship between the 2 sets of SNP weights was approximately linear.

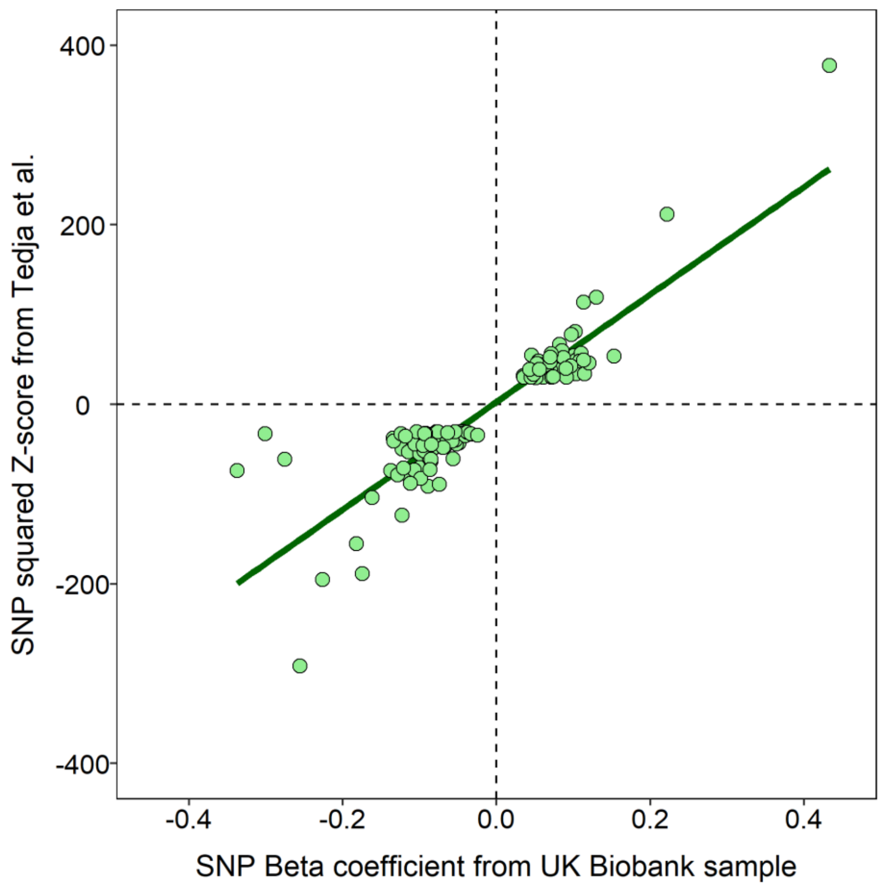

Supplementary Figure S3. Estimated causal effect of refractive error on years spent in education (EduYears) obtained using non-linear Mendelian randomization in a leave-one-out analysis. The analysis reported in Figure 3B of the main text was repeated 128 times, each time excluding one of the 129 genetic variant instrumental variables. Error bars are the 95% confidence interval.

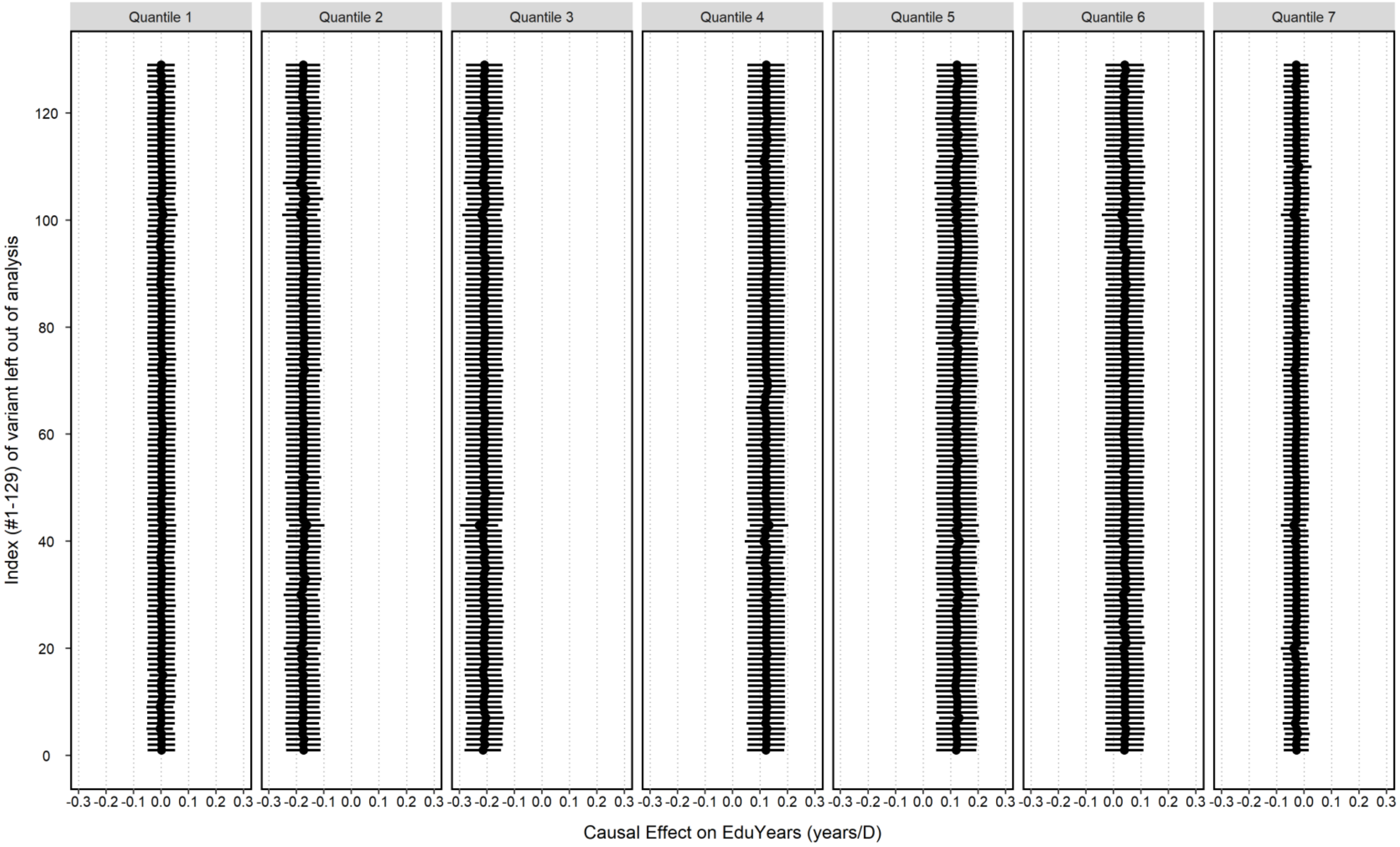

Supplementary Figure S4. Estimated causal effect of refractive error on years spent in education (EduYears) obtained using non-linear Mendelian randomization without adjustment for covariates. The analyses reported in Figure 3 of the main text were repeated without adjustment for covariates (age, age^2, age^3, gender, Northing, Easting, genotyping array and the first 10 ancestry genetic principal components). Error bars are the 95% confidence interval.

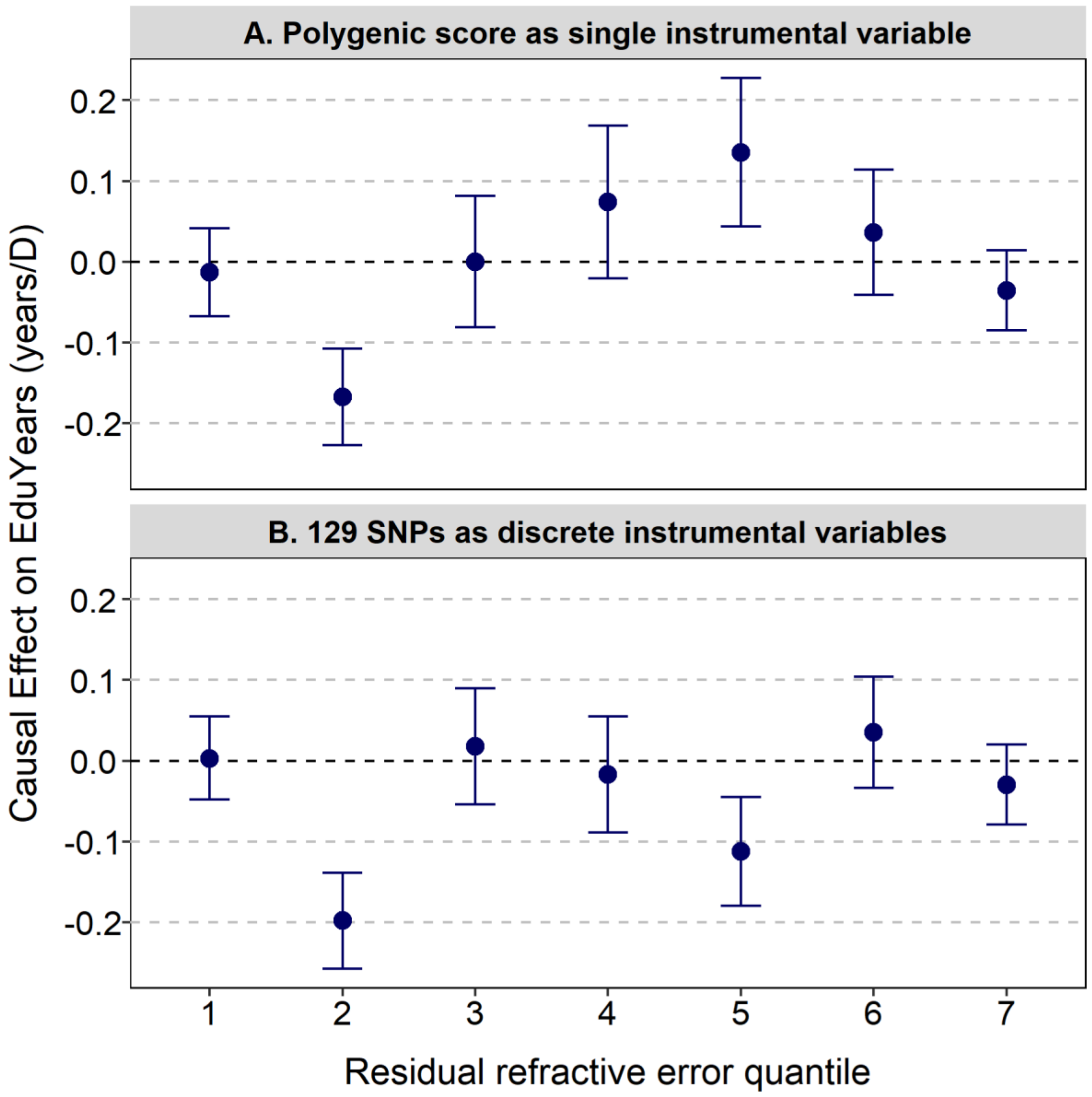

Supplementary Figure S5. Confounding bias plot. Relative bias in instrumental variable (IV) estimate vs. ordinary least squares (OLS) regression estimate from potential confounders. Points show the bias in units of standard deviations of the confounder variable per 1 standard deviation change in the original or instrumented exposure variable (refractive error). Error bars are the 95% confidence interval. Townsend deprivation index (TDI) was natural log transformed to reduce skew.

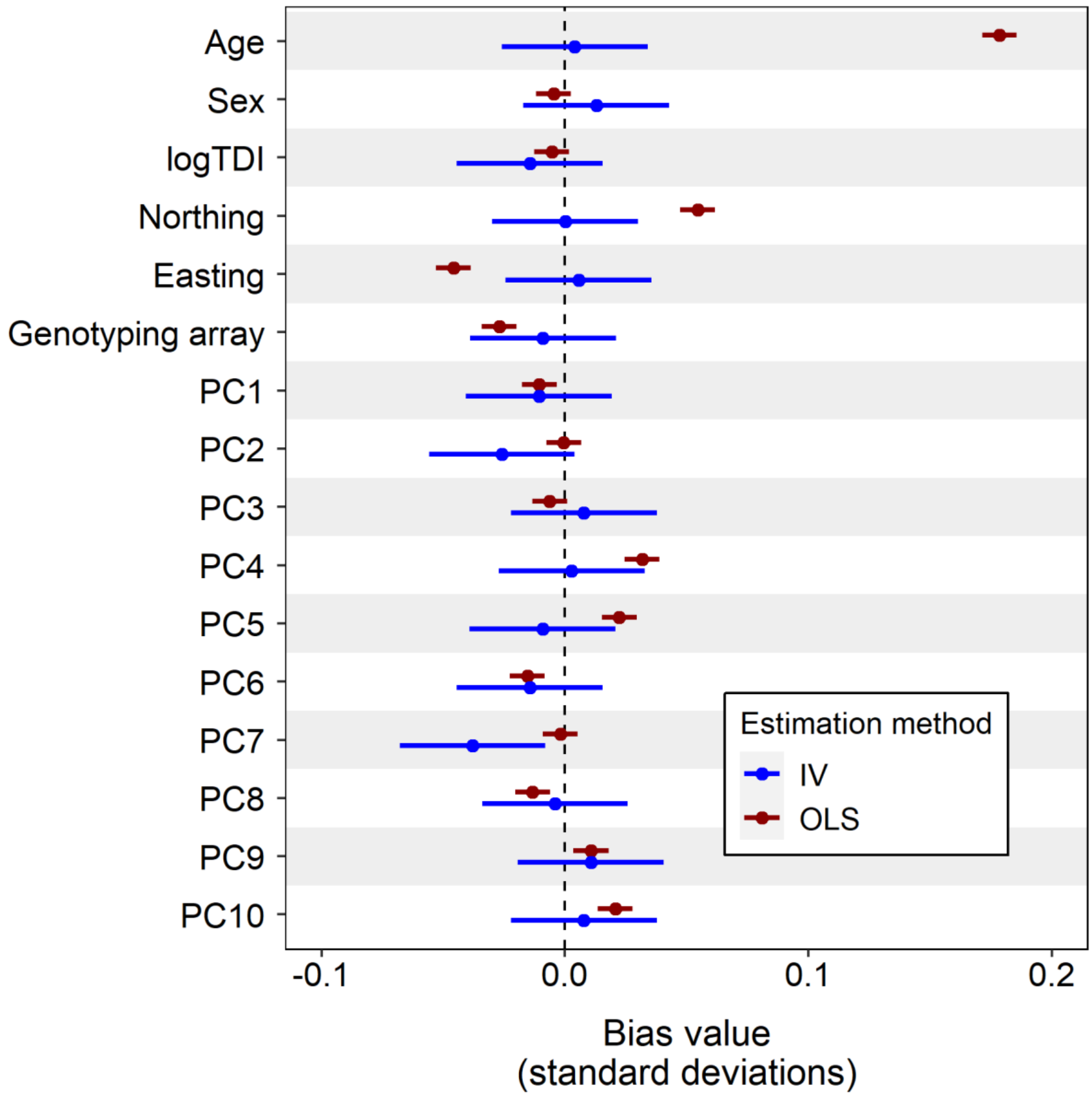

References

1. Tedja MS, Wojciechowski R, Hysi PG, et al. Genome-wide association meta-analysis highlights light-induced signaling as a driver for refractive error. *Nature Genetics* 2018;50:834-848.

2. Ghorbani Mojarrad N, Williams C, Guggenheim JA. A genetic risk score and number of myopic parents independently predict myopia. *Ophthalmic Physiol Opt* 2018;38:492–502.
